# Prevalence of female genital mutilation and its effect on women’s health in Bale zone, Ethiopia: a cross-sectional study

**DOI:** 10.1186/1471-2458-14-1076

**Published:** 2014-10-16

**Authors:** Daniel Bogale, Desalegn Markos, Muhammedawel Kaso

**Affiliations:** Department of Public Health, College of Medicine and Health Sciences, Madawalabu University, Bale, Goba, Ethiopia; Department of Nursing, College of Medicine and Health Sciences, Madawalabu University, Bale, Goba, Ethiopia

## Abstract

**Background:**

Females’ genital mutilation (FGM) is one of the harmful traditional practices affecting the health of women and children. It has a long-term physiological, sexual and psychological effect on women. It remains still a serious problem for large proportion of women in most sub-Saharan Africa countries including Ethiopia.

**Methods:**

A community based cross sectional study design which is supplemented by qualitative method was conducted in 2014. A total of 634 reproductive age women were involved in the quantitative part of the study. The respondents were drawn from five randomly selected districts of Bale zone. The total sample was allocated proportionally to each district based on the number of reproductive age women it has. Purposive sampling method was used for qualitative study. Then, data were collected using pre-tested and structured questionnaire. The collected data were analyzed by SPSS for windows version 16.0. Multiple logistic regressions were carried out to examine the existence of relationship between FGM and selected determinant factors. Variables significant in the bivariate analysis were then entered into a multiple logistic regression analysis.

**Results:**

In this study, 486 (78.5%) of women had undergone some form of FGM with 75% lower and 82% upper confidence interval. To get married, to get social acceptance, to safeguard virginity, to suppress sexual desire and religious recommendations were the main reasons of FGM. The reported immediate complications were excessive bleeding at the time of the procedure, infection, urine retention and swelling of genital organ. Muslim women and women from rural areas were significantly more likely to have undergone the procedure. In addition to these, compared to women 15–20 years old older women were more likely to report themselves having undergone FGM.

**Conclusions:**

Although younger women, those from urban residence and some religions are less likely to have had FGM it is still extremely common in this zone. Deep cultural issues and strongly personally held beliefs which are not simple to predict or quantify are likely to be involved in the perpetuation of FGM. Efforts to eradicate the practice should incorporate a human rights approach rather than rely solely on the damaging health consequences.

## Background

Females’ Genital Mutilation is refers to "all procedures that involve partial or total removal of the external features of the female genitalia or other injuries to the female genital organs whether for cultural or other nontherapeutic reasons [1]. It involves surgical removal of parts or all of the most sensitive female genital organs [2]. As defined by the World Health Organization [3, 4], there are three types of FGM: Type I: Consists of excision of the clitoral prepuce, and it is known in Muslim countries as the Sunnah circumcision. Type II: Involves excision of the prepuce, the removal of the glans clitoris or the entire clitoris with varying degrees of the adjacent parts of the labia minora. This is often referred to simply as excision. Type III: Termed infibulation, and also referred to as Pharaonic circumcision. It is the most drastic procedure and the one resulting in the most serious adverse health effects. It involves removal of the whole clitoris, the whole of the labia minora and the medial parts of the labia majora. The latter structures are then approximated with thorns, catgut or silk sutures (depending on the operator). This results in the occlusion of the genitalia leaving only a small hole to allow the passage of urine and menstrual blood.

The practice of FGM has been identified as being performed in many regions all across the world. It is primarily practiced among various ethnic groups in more than 28 countries in Africa [5]. The practice is deeply rooted and heavily prevalent mostly in the countries that have a strong connection to the Islamic religion [6]. Various pocket studies from different countries report different level of FGM. Kersa district (Ethiopia) (92.3%) [7], Sierra Leone (81.2%) [8], Mauritania (77%) [9], Gambia (75.6%) [10], Rural Gambia (58%) [11], Ravansar (Iran) (55.7%) [12] and Nigeria (34%) [13]. Somalia (98%), Djibouti (93%), Eritrea (89%) and Ethiopia (74%) are east African countries where FGM is widely spread [1]. The distribution of FGM practice in Ethiopia is vary depending on ethnic origin and region [7].

It is clear that tradition, religion and social pressure are the main motives for performing FGM [12]. In some of the ethnic groups FGM is compulsory, while in others, women who have not undergone the practice may find it difficult to get married [2]. Respondents from countries where FGM is traditionally performed believed that FGM is sanctioned by Islamic religion. Individuals who did not conform to the practice were considered to be acting against their religion and the Qur’an [5]. An act of obedience or honour to the teachings of religion, ensuring that improper sexual conduct amongst the females is kept at a minimum, for societal approval, and increasing the sexual satisfaction of the males [6]. FGM forms an important part of the rites of passage ceremony, marking the coming of age of the female child [2]. It is deeply embedded in society, and its elimination requires a clear understanding of the cultural perceptions and beliefs it feeds on [14].

The practice takes place without the administration of anesthetics and under very unhygienic conditions. Mixtures of local herbs, earth, cow dung, ash or butter are used to treat the wound [2]. The reported immediate complications were excessive bleeding at the time of the procedure, difficulty with urination, shock during the procedure [2, 13], ulceration of the genital region and injury to adjacent tissue [2]. Fibrosis, keloids, synechia, and clitoral neuroma are consequences of FGM, and that their prevalence rises progressively from type I to type II [10]. Deliveries to women who have undergone FGM are significantly more likely to be complicated by caesarean section, postpartum haemorrhage, episiotomy, extended maternal hospital stay, resuscitation of the infant, and inpatient perinatal death, than deliveries to women who have not had FGM [4]. Prolonged labor is significantly increased in women who had undergone type I or II FGM [10]. Women with FGM type II and type III were significantly more likely to have a caesarean section and postpartum blood loss than women who had not had FGM [4]. Difficult penetration during intercourse [10] is also another long term consequence of FGM.

Females’ genital mutilation violates human rights [3]. It is estimated that around 140 million girls and women worldwide have undergone FGM and that at least two million girls are annually at risk of undergoing some form of the procedure [14]. Despite the banning of the practice within countries, its eradication is still a problem as the practice merely gets hidden from the eyes of the public and continues quietly [6]. Although the achievement so far has not been as desired, Ethiopia has made an important progress towards the reduction of FGM. According to the 2000 and 2005 Demographic and Health Survey (DHS) reports of Ethiopia, the prevalence rates for FGM were found 80% and 74%, respectively [15, 16].

To achieve success in preventing the continuation of FGM, it is necessary to understand the forces underpinning the practice such that information, messages, and activities can be tailored to their audiences accordingly. Therefore, this study was intending to assess the current prevalence of FMG, its health consequences and factors underpinning the perpetuation of this practice.

## Methods

The study was conducted in Bale zone from April 18, 2014 to May 20, 2014. It is one of the 18 rural zones of Oromia regional state located in Southeast of Ethiopia. It is the second largest zone in the region with an area of 67,329.6 km^2^ that extends from 5^0^ 22’- 8^0^ 08’N latitudes and 38^0^ 41’- 40^0^ 44’E longitudes and having 14.93% highland, 21.54% midland and 63.55% lowland distribution [17]. Eighty percent of the zone is composed of farmers and agro pastoralists. There are 10 farmers, 8 agro pastoralists and 2 town administrative districts in the zone. The composition of rural and urban population is 87.5% and 12.5%, respectively. According to 2007 census Bale zone has a total of 1,418,864 population out of which 697,185 were females [18]. A community based cross-sectional study was employed. To address the perceptional, cultural and practical aspects of FGM, qualitative methods was employed and presented by triangulating the findings obtained by quantitative results.

All Bale Zone child bearing age women were the source population for this study. For quantitative study all child bearing age women found in five randomly selected districts namely, child bearing women of Agarfa district, Dinsho district, Madawalabu district, Berbere district and Robe town were the study population. The study subject was randomly selected reproductive age women from aforementioned districts. For qualitative study the same districts were used to select women for Focus Group Discussion (FGD) and in-depth interviews.

The study employed single population proportion sample size determination formula. Proportion of FGM in the study area was assumed 50%. Even if there were few similar studies conducted in the country, they were representing homogenous study subjects from a single district. However, the current study addressed the study subjects of various social and cultural compositions. These are the reasons why the authors preferred to consider this assumption. Ninety five percent confidence level with 5% margin of error was considered. To minimize the effect of stratification on the result that would be obtained through simple random sampling 1.5 design effect was considered and 10% of the total sample was added for non response rate. Finally, the final total sample size was 634 child bearing age women.

The sampling procedure for quantitative study was first, 20 districts were stratified in to three groups based on their livelihood. The three strata were agrarian, pastoralist and town administrative districts. The assumption behind this stratification was; there is difference in accessibility of information and composition of society in different districts. Then, one district from town administrative stratum and two districts from agrarian and two districts from pastoralist (a total of five districts) were selected randomly from the list of each stratum. Finally, the total sample size was proportionally allocated to five districts after the number of reproductive age women were determined. Three kebeles (the smallest administrative unit) were selected from each district through simple random sampling. After the number of women in that kebele was determined, sample size allocated for each district was allocated again to each kebele. Finally, women in that kebele were selected by simple random sampling method. For qualitative study, we used a purposive sampling strategy to ensure diverse respondents (different age group and different role in their community) and geographic representation (rural, urban, pastoralist and agrarian) of key perspectives in FGM practice. This sampling method was employed both for in-depth interview and FGD. Authors thought there were issues a woman might not be discussed in a group freely because this practice is highly tied with cultural and social values. The problem a woman encountered might be better explained in an in-depth interview. FGDs having on average 8 participants were arranged on purposively selected kebeles. FGDs were considering both rural and urban settings. A total of four FGDs and 8 in-depth interviews were made with purposively selected women. The numbers of FGDs were determined by the ideas generated from the discussion. Similarly, the redundancy (saturation) of ideas obtained from in-depth interviews fixed to eight.

Structured and standardized tool [19] is used after it was translated in to the local languages (Amharic and Afan Oromo). The English version of the tool was translated to local languages by a person who is fluent in English, Amharic and Afan Oromo languages. Again both Amharic and Afan Oromo version tools were translated back to English by another person with similar ability to check for its original meaning. Then, it was pretested on 5% of similar population on one of the district which was not included in the final sample. Findings and experiences from the pre-test was utilized in modifying the tool.

Eight diploma nurses who are fluent in speaking Amharic and Afan Oromo were involved in the data collection process. All data collectors and supervisors were trained for two days on data collection process. The principal investigator and the supervisors strictly followed the overall activities of data collection on daily bases to ensure the completeness of questionnaire, to give further clarification and support for data collectors.

Homogeneous purposive sampling method was employed to select discussants for FGD and in-depth interview. The groups for FGD were made homogeneous in terms of age, the role they had in the community and economic status. Non-sensitive issues were addressed by FGDs. Nonthreatening environment and easily accessible location were arranged to keep the privacy of the participants. These helped the participants to talk freely whatever they know and feel. In the same way, perceptions, experiences, cultural and practical aspects of FGM were discussed with purposely selected women. Validity was ensured through transcription of the local language in to text from the tape recorder by two data collectors and translation to English language was made by two researchers simultaneously.

The data were checked for completeness and consistencies then, cleaned, coded and entered in to computer using statistical package for social sciences (SPSS) windows version 16.0. A point and interval estimate of prevalence of FGM was described. Additionally, binary and multiple logistic regression analyses were carried out to examine the existence of relationship between practice of FGM and selected independent variables. Variables having P-value of less than 0.05 on binary logistic regression were the candidate for multiple logistic regressions. Statistical significance was declared at P < 0.05. For qualitative part, data were transcribed into local language text by the data collectors by replaying the recorded interview and translated into English language by the two researchers. Different ideas in the text were merged in their themes and a thematic analysis was employed manually by the investigator. Then, result was presented in narration by triangulating the quantitative findings.

The proposal was approved by ethical review committee of Madawalabu University. Furthermore, letter of permission was obtained from Bale zone administrative and health office and from each district administrative and health office. Verbal consent was obtained both from quantitative and qualitative study subjects after the study objectives, procedures and their right to refuse participation and dropping interview any time they want were assured. For this very purpose, a one page consent letter was attached as the cover page of each questionnaire. The qualitative part of this manuscript complies with the RATS guidelines for reporting qualitative research [20].

## Results

### Socio-demographic characteristics of the study participants

Out of 634 reproductive age women who were planned for the study, 619 were successfully interviewed yielding the response rate of 97.6%. The mean age of the respondents was 30 (SD ± 9.2). More than sixteen percent of respondent, 101(16.3%), were between the age group of 15 and 20 years. Muslim was found a predominant religion which accounts 366(59.1%) followed by Christian orthodox, 36.5% (Table 1).

**Table 1:** **Socio-demographic characteristics of the respondents, Bale zone, Oromia region, South-East Ethiopia, May, 2014**

Characteristics	Frequency	Percent
**Age of respondent**		
15-20	101	16.3
20-25	163	26.3
26-30	128	20.7
31-35	57	9.2
36-49	170	27.5
**Marital status**		
Single	6	1
Married	574	92.7
Divorced	25	4
Widowed	14	2.3
**Religion**		
Muslim	366	59.1
Orthodox	226	36.5
Protestant	27	4.4
**Ethnicity**		
Oromo	540	87.2
Amhara	79	12.8
**Respondents’ educational status**		
Unable to read and write	229	37
Read and write	9	1.5
1^0^ education	233	37.6
2^0^ and above education	148	23.9
**Husband education**		
Unable to read and write	129	22.5
Read and write	25	4.4
1^0^ education	206	35.9
2^0^ and above education	214	37.3
**Respondents’ occupation**		
House wife	448	72.4
Gov Employee	33	5.3
Self employee	10	1.6
Farmer	24	3.9
Merchant	84	13.6
*Other	20	3.2
**Residence**		
Rural	129	20.8
Urban	490	79.2

*Students and daily labor.

Greater than one third of the participants, 233 (37.6%), reported that they did attend primary level education which is almost as equal as those who did not able to read and write, 229 (37%). One hundred twenty nine (22.5%) husbands of the study subjects were unable to read and write where us 114 (37.3%) of them were secondary and above educational level (Table 1).

Majority, 540 (87.2%) of the respondents were ethnically Oromo and the vast majority, 574(92.7%), of the study subjects were married while only 6 (1%) women were single. Large number, 448 (72.4%) of study subjects were house wife (Table 1).

### Respondents’ source of information

More than half, 384 (62%) of the respondents had radio as a means of source of information while 244 (39.4%) of them had TV. Magazine was mentioned by, 195 (31.5%) study subjects as a means of source of information.

### Prevalence and types of FGM

Almost all, 609 (98.4%) of the study the study subjects had heard of FGM. However, only 246 (39.7%) knew the practice is performed in their community right during the study period. In this study, 486 (78.5%) of women had undergone some form of FGM with 75% lower and 82% upper confidence interval. The mean age of the study participants during circumcision was 7.89 (SD ± 4.56).

The most prevalent form of FGM study subjects and their last (young) daughters undergo was partial or total removal of clitoris (type II). Four hundred forty one (78.6%) of the study subjects and 131 (87.3%) of their last (young) daughters were undergo this form of procedure. Genital area sewn closed after mutilation of some parts of genital was the second larger procedure which accounts 44 (7.8%) among the study subjects and 12 (8%) among their last (young) daughters (Table 2).

**Table 2:** **Reported anatomical description of FGM among respondents and on their last daughter in Bale zone, Ethiopia, 2014**

Anatomical description	Frequency	Percent
**On respondents**		
Flesh removed (partial or total clitoris removed) (type II)	441	78.6
Genital area sewn closed after mutilation of some parts of genital (type III)	44	7.8
Genital area pricked/nicked (type I)	14	2.5
Don’t know	62	11.1
**On last (younger) girl**		
Flesh removed (partial or total clitoris removed) (type II)	131	87.3
Genital area sewn closed after mutilation of some parts of genital (type III)	12	8.0
Genital area pricked/nicked (type I)	7	4.7

### Reasons for the practice of female genital mutilation

Some of the reasons given by the study subjects for the practice of FGM and its perpetuation were; to get married, 100 (78.7%), to get social acceptance, 95 (74.8%) and to safeguard virginity, 87 (68.5% (Table 3). These findings were supported by the qualitative part this study.

**Table 3:** **The common reasons given by the study subjects for the practice of FGM, Bale zone Ethiopia, 2014**

Reasons	Frequency	Percent
To get husband	100	78.7
To get social acceptance	95	74.8
To safeguard virginity	87	68.5
To suppress sexual desire	54	42.5
Religion rite	41	33.3
To comfort for male during sex	14	11

Note: a respondent might give multiple answers for the reason of FGM that is why the sum of the percent exceeded 100.

"*In our religion, anybody reaching 15 years of age be it boys or girls are expected to have ‘Sollat’ (praying) five times a day. This is possible for a girl if and only if she undergo circumcision. Her ‘Sollat’ is not accepted by Allah unless she is circumcised.* She was a 45 years old woman from pastoral community.

Another 34 years old pastoral woman said, "*Leave everything, who is going to touch her flesh when she die? Even religious fathers will not pray on her flesh. You see how much it is shame for her family?*"

The recent study identified that both mothers and fathers, 356 (57.5%) are decision makers for their daughter to undergo circumcision. Many respondents, 231 (37.3%) identified mothers are the only decision makers for a daughter to be circumcised. The majority of the respondents, 523 (84.5%) mentioned that the circumcisers are females. Traditional circumcisers, 474 (76.6%), old age people, 42 (6.8%) and traditional birth attendants, 10 (1.6%) were identified by the study participants as an operators of FGM in the study area. The rest, 93 (17%) of the respondents did not know the person performing the procedure (Table 4).

**Table 4:** **Decision makers, circumcisers and sex of circumcisers in Bale zone, Ethiopia 2014**

Characteristics	Frequency	Percent
**Decision makers**		
Mother	231	37.3
Father	10	1.6
Both mother and father	356	57.5
Others	21	3.5
**Circumcisers**		
Old age people	42	6.8
Traditional circumciser	474	76.6
TBA	10	1.6
Don’t know	93	15
**Sex of circumcisers**		
Female	523	84.5
Male	24	3.9
Both sex	72	11.6

Others include: a girl herself, grandparents, other relatives and neighbors.

FGM has different prociders and reveald by the qualitative part of this study.

"*I was 13 years old when my mother told me to be ready for circumcision; actually I knew I should be circumcised bacause all of my friends were under go the procedure. One day morning three women were in our home when I came home back from river. Immediately, my mother told me to wait here in the hut behind the big house. Then, the three women and my mother came to me and they told me to lay back on the floor. One woman sit on my chest and caught both of my hands down to the ground, my mom and another woman pulled my legs apart. Then, don’t ask me what happened to me*" she was laughing and nodding head. A 32 years old rural woman.

Another 49 years old woman explained the procedure as follow: "*Now every thing is simple. When we were young, there were big ceremony during circumcision. A family prepare a goat, flour, milk and honey for a girl to eat at least for a week after procedure. There is a wood known as ‘Qumbi’ it is grinded and put on the new wound of the circumcised genital organ and then the blood from slaughterd goat will be poured on the wound. The blood together with this wood help a wound to heal and keep the oppening narrow*".

*"….. if a girl under going the procedure can urinate easily with in three days after the procedure, it is assumed not well performed. Then, it will be performed again by the same woman"* 45 years old pastoralist woman.

### Short term consequences of FGM

**Figure 1:**
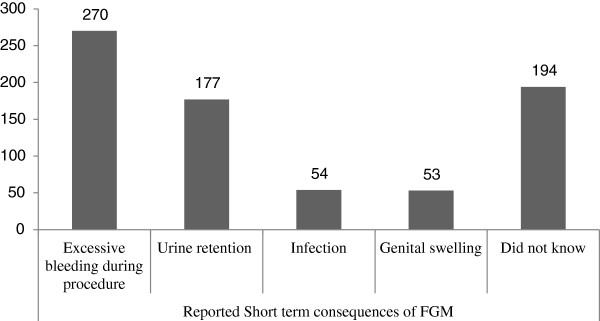
**Reported short term consequence of FGM among the study subjects in Bale zone 2014.**

In the current study FGM related short term health problems were assessed among the study subjects. Out of these consequences; excessive bleeding during the procedure, 270 (55.8%), urine retention, 177 (36.6%), infection, 54 (11.1%) and swelling of genital, 53 (11%), were problem encountered and reported by the respondents (Figure 1).

This circumcision related immediate health problems were assessed by qualitative part of the study. A 25 year grade 2 attending in-depth interview participant woman reported that, *‘I circumcised when I was 12 year old. I do not forget that moment. Three women, they were my mothers’ friends sitting on my hands and legs and one of the old woman slaughtered me. Oh my God, it was painful. I suffered to urinate for five days. The wound was not dry for a month’*.

Another young in-depth interviewee woman said that, ‘*There was a woman in this village who circumcised most of girls. In the same way I was circumcised when I was ten years old. This woman was very old. During the procedure I cannot tolerate the pain so that I moved here and there to skip the pain. When the procedure completed, I cannot manage myself; I was shocked. When I come to normal condition, the blood was not only from my genital organ but also from the upper part of my left leg. I thought that woman cut my leg when I moved here and there during the procedure*’.

### Self reported long term consequences of FGM

**Figure 2:**
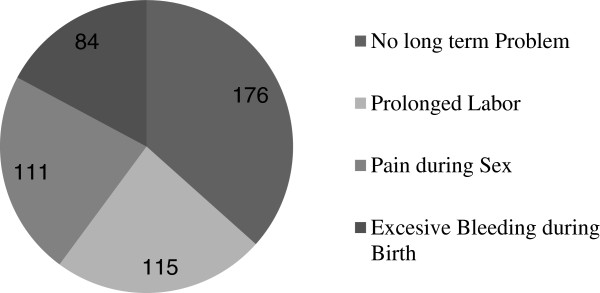
**Reported long term consequence of FGM among the study subjects in Bale zone 2014.**

Out of 486 (78.5%) circumcised study participants, 180 (37%) were reporting long term health problems they encountered because of FGM. The reported long term problems were prolonged labor, 115 (37.1%), pain during sexual intercourse, 111 (35.8%) and excessive bleeding during birth, 84 (27.1%) (Figure 2).

Long term health problems related to this practice were also assessed through in-depth interview and FGD. ‘*I have no sense during sex rather I fill pain. There is big scar resulted from circumcision. When I delivered my first child, the labor was too long; three days. I prefer not to talk what happened to me. My blood was like a blood from slaughtered animal’*. A 34 year old 8 grade attended urban resident of in-depth interview participant.

Another 40 year old woman that did not attend any formal education reported that, ‘*I put one of my last daughter under circumcision by a well known circumciser found in another kebele when she was 1 year old. At that time I didn’t see any problem except a little bleeding. One day after a year I was washing her body. At this time I observed a strange thing around her genital organ. It had no normal anatomy of external reproductive organ of a female. Except small opening for urination, the limps of her external reproductive organ were stitched together. I thing the blood during circumcision was dried around the limps of her genital organ and close the opening. What I did is immediately I went to that woman and consulted her. She ordered me to bring my daughter to her home. She saw her reproductive organ and told me to bring blade. Then, she tore the closed anatomy and made it normal. I was scared that my daughter may suffer during labor but thanks to God, she delivered safely’*.

### Factors associated with FGM/C

After doing binary logistic regression, controlling the possible confounder multiple logistic regressions were computed to explore the association between selected independent variables and female genital mutilation/cutting.

The odd of circumcision is greater than three times more likely among rural resident when compared with urban counterparts (AOR = 3.31, 95% CI: 1.48-7.43). Additionally, age of the respondents was statistically significant with FGM. As compared to 15-20 age category of study subjects odds of circumcision among 26-30, 31-35 and 36-45 age category were (AOR = 2.23, 95%CI: 1.12-4.42), (AOR = 3.93, 95%CI: 1.46-10.62) and (AOR = 13.46, 95%CI: 5.14-35.25) times higher, respectively. Concerning religion, as compared to protestant Muslims were circumcised 3.55 times more likely (AOR =3.55, 95%CI: 1.35-9.37) (Table 5).

**Table 5:** **Factors associated with FGM among the respondents, Bale zone, Oromia region, South-East Ethiopia, May, 2014**

Variable	Circumcised		COR (95% CI)	AOR (95% CI)
	Yes	No		
**Residence**				
Rural	121 (93.8%)	8 (6.2%)	5.18 (2.46, 10.89)**	3.31 (1.48, 7.43)**
Urban	365 (74.5%)	125 (25.5%)	1	1
**Age**				
15-20	66 (65.3%)	35 (34.7%)	1	1
21-25	108 (66.3%)	55 (33.7%)	1.04 (0.62, 1.76)	1.29 (0.71, 2.33)
26-30	100 (78.1%)	28 (21.9%)	1.89 (1.05, 3.40)*	2.23 (1.12, 4.42)*
31-35	49 (86%)	8 (11%)	3.25 (1.39, 7.62)*	3.93 (1.46, 10.62)**
36-49	163 (95.9%)	7 (4.3%)	12.35 (5.22, 29.19)**	13.46 (5.14, 35.25)***
**Religion**				
Muslim	306 (83.6%)	60 (16.4%)	3.5 (1.55, 7.93)**	3.55 (1.35, 9.37)**
Orthodox	164 (72.6%)	62 (27.4%)	1.8 (0.8, 4.14)	1.65 (0.61, 4.40)
Protestant	16 (59.3%)	11 (40.7%)	1	1
**Respondent Education**				
Unable to read and write	214 (89.9%)	24 (10.1%)	5.27 (3.08, 9.03)*	2.04 (0.97, 4.27)
1^0^ education	179 (76.8%)	54 (23.2%)	1.96 (1.25, 3.08)*	1.34 (0.72, 2.44)
2^0^ education	93 (62.8%)	55 (37.2%)	1	1
**Husband Education**				
Unable to read and write	143 (92.9%)	11 (7.1%)	3.53 (1.75, 7.09)***	1.82 (0.79, 4.19)
1^0^ education	162 (78.6%)	44 (21.4%)	5.92 (3.0, 11.67)***	0.93 (0.54, 1.61)
2^0^ education	147 (68.7%)	67 (31.3%)	1	1
**Occupation**				
House wife	363 (81%)	85 (19%)	1.56 (1.01, 2.44)*	1.21 (0.71, 2.04)
Government employee	22 (66.7%)	11 (33.3%)	0.73 (0.32, 1.66)	0.86 (0.31, 2.33)
Self employee	101 (73.2%)	37 (26.8%)	1	1

Note: *Significant at P <0.05, **significant at P <0.01, ***significant at P <0.001.

## Discussion

Females’ genital mutilation is among the traditional practices which are not only prejudicial and harmful to the life of a child but also discriminatory against to the girl child [2]. In this study, the reported prevalence of FGM and its different forms was assessed among reproductive age women. In order to get a good understanding of its current practice in the area, this study also tried to assess the types of FGM committed on the last (youngest) daughters of respondents if they put them under the procedure.

Accordingly, the prevalence of FGM in this study was 78.5% which is slightly higher than the prevalence from 2005 Ethiopian Demographic and Health Survey (EDHS) report, 74% [16]. The current study represents only one region of the country where there is higher level of FGM practice. However, the EDHS report represents different parts of the country where the magnitude of this practice is vary. The finding of this study also compared with the study conducted in Kersa district (92.3%) [7]. The possible justification could be the previous study represents only one district in which FGM might be practiced widely. However, the current study represented town, agrarian and pastoralist areas where there is various level of FGM. It was also compared with the study conducted in other countries like Sierra Leone (81.2%), [8] Mauritania (77%), [9] Gambia (75.6%), [10] which had almost similar findings.

In this study using the World Health Organization classification of FGM, 78.6% of the respondents reported that they undergone procedure type II. Similarly, the study conducted in Nigeria showed that most commonly practiced FGM was type II [13]. Type III had a lower prevalence, 7.8% which was almost as equal as the finding from Gambia [21]. However, magnitude of type III FGM in this study was much lower than another study in which 36% of vaginal stitching (type III) operation [22]. The lower proportion of infibulations (type III) in our sample might also be attributable to under reporting of the procedure. Even if the prevalence of type III FGM is lower in this study, the procedure is sever and putting woman’s health under risk. Eight percent of the last (youngest) daughters of the study subjects were also under gone this risk full procedure.

Many people do not easily understand the distinctions between the religion, culture and the practice of FGM [22]. In the current study various reasons were given for the perpetuation of FGM practice in the study area. To get married, to get social acceptance, to safeguard virginity, to suppress sexual desire and religious recommendations were the main reasons for performing FGM. Similarly, in other study keeping traditions, cleanliness, religious recommendations and sexual desire control were the reasons for performing FGM [12]. Respondents in this study have attested that FGM is a practice that the religion claims is a right for the women. They tend to view the practice as a religious obligation mainly related to the Islamic religion, which is the prevalent religion in the study area. Similar finding was obtained from the study conducted in Eastern Ethiopia, religion has been found to be one of the main motives for performing FGM among Muslim community [23].

FGD participants also explain the reason for the perpetuation of FGM and perceived that *uncircumcised woman is not pure and clean in the eyes of God*. This practice also observed from the perspectives of marriage ability of a girl and her sexual desire control. Study participants have attested that ‘*no one considers uncircumcised girl in the community for marriage. The burden is not only for the girl but also for her family. A girl should be undergoing circumcision to be managed for her husband unless he cannot satisfy her sexual desire. In this case, she goes out to see for other man to satisfy her sexual desire’*.

Most of the study participants reported the operation is performed in their communities by traditional operators, local old age people and traditional birth attendants. Similarly, in other study herbalist, traditional birth attendants, parents, and relatives were the operators [13]. However, in the study conducted in rural Gambia all operations were undertaken by traditional operators [11]. This could be due to difference in operational definition for the operators in different countries. In this study the majority of FGM operations is performed by female circumciser which was in line with the study conducted in Iran [12]. Regarding the decision for FGM, 57.5% of the respondents mentioned that their mothers and fathers were responsible in making the decision. In other study mothers and grandmothers were the dominant decision makers [12]. The report from in-depth interview revealed that all the family members are responsible for a girl to be circumcised. *‘Even if only a mother presents on an operation, a father also facilitate things before hand’.*

The reported immediate complications were excessive bleeding at the time of the procedure, infection, urine retention and swelling of genital organ. These complication also reported from the study conducted in Nigeria, excessive bleeding at the time of the procedure, difficulty with urination and having collapsed [13]. Another study from Gambia also showed the most common immediate complication for all types of FGM was infection which is associated in some cases with haemorrhage and anaemia [21]. Similarly, severe pain, shock, haemorrhage, urine retention, ulceration of the genital region and injury to adjacent tissue were immediate complications [2]. The long term consequences of this practice were also assessed in the qualitative study especially, in the in-depth interview. ‘*A woman loss her sexual desire completely. Once she is circumcised, her role during sexual intercourse will be observing the phenomenon’.*

Deliveries to women who have undergone FGM are significantly more likely to be complicated by caesarean section, postpartum haemorrhage, episiotomy, extended maternal hospital stay, resuscitation of the infant, and inpatient prenatal death, than deliveries to women who have not had FGM [4]. In the current study prolonged labor, pain during sex and excessive bleeding at the time of birth were the reported long term consequences of FGM. Some of these consequences were reported in the qualitative part of this study. *‘Some women hate the night because of a pain during sexual intercourse. God created a person equally and allowed to enjoy things equally but… the challenges are many for a woman’*.

When tested, significant association was found between residence, religion and age of the respondents. Muslim women and women from rural areas were significantly more likely to have undergone FGM which was similar finding from Burkina Faso [24]. In addition to these, compared to women 15–20 years old older women were more likely to report themselves having undergone FGM. Interestingly, the study conducted in Mauritania showed that older women (ages 45–49 years old) were less likely to report experiencing FGM compared to women 15–19 years old [9]. This difference could be due to cultural variation in reporting this practice and the difference in the study subjects. On the other hand, educational level of the respondents was statistically significant only at a bivariate level but it was determinant variable in other study conducted in Iraq [12]. The possible justification could be this practice has strong tie with social and cultural values in the study area what might not change easily.

While the large sample size as well as the mixed study design approach constitutes a major strength for this study, it also has some limitations. Considering that FGM is a very sensitive and stigmatizing social issue in the study area, this leaves room to question the truthfulness of a respondent when questioned by an unknown interviewer. The likelihood for women to give culturally acceptable answers to the interviewer constitutes a real concern.

## Conclusions

FGM is still practiced in Bale zone of Ethiopia and resulted in various forms of damage/injury on more than three in four women of reproductive age. Although younger women, those from urban residence and some religions are less likely to have had FGM it is still extremely common in this zone. Deep cultural issues and strongly personally held beliefs which are not simple to predict or quantify are likely to be involved in the perpetuation of FGM. Efforts to eradicate the practice should incorporate a human rights approach rather than rely solely on the damaging health consequences. For Muslim women most benefits are likely to be gained from working with religious groups and leaders. As this practice intricate with culture and beliefs, interested researchers are recommended to dig out the driving forces perpetuating this practice and intention for its continuation.
